# Fabrication of CPW-Fed Fractal Antenna for UWB Applications with Omni-Directional Patterns

**DOI:** 10.1155/2014/391602

**Published:** 2014-02-03

**Authors:** Tohid Sedghi, Mahdi Jalali, Tohid Aribi

**Affiliations:** ^1^Department of Electrical Engineering, Urmia Branch, Islamic Azad University, Urmia 5717114653, Iran; ^2^Department of Electrical Engineering, Naghadeh Branch, Islamic Azad University, Naghadeh, Iran; ^3^Department of Electrical Engineering, Miandoab Branch, Islamic Azad University, Miandoab, Iran

## Abstract

Novel and compact CPW-fed antennas are proposed comprised of a fractal patch and modified ground-plane. The ground-plane is truncated at the center and includes dielectric notches at its side to enhance the antenna's impedance bandwidth. The dimensions of the notches effectively control the upper and lower band edges of the antenna. The optimized antenna operates across 2.95–12.81 GHz for *S*_11_ ≤ −10 dB. Omnidirectional radiation pattern is achieved over the full UWB frequency range. The miniaturized antenna has a total size of 14 × 18 × 1 mm^3^. The characteristics of the proposed antenna are suitable for UWB wireless communication requiring low profile antennas.

## 1. Introduction

Rapid development of wireless systems, a lot of concentration is being given for designing the UWB antennas, since they are the key elements to radiate and receive the signals [1]. There has been great progress in the design of ultrawideband antennas and devices in recent years. Several impedance alteration techniques are mentioned which vary with wide rectangular slots and circular slots with hexagonal forms [2]. One of the major challenges in the design of ultra wideband antennas is how to achieve small size antennas with low weight and desired radiation pattern characteristics and electrical properties in the band of interest [3]. The numerous numbers of slots cause more bandwidth and the optimum feed structure gives the good impedance matching [4]. Recently, some coplanar waveguide- (CPW-) fed printed monopole antennas have been reported [5–7]. This technology has offered unique advantages not achievable by conventional narrowband technology, which includes low power requirements, high speed transmission, immunity to multipath propagation, and simple hardware configuration. UWB is targeted as a cable replacement technology. Applications include wireless home networking, high-density use in business cores, wireless speakers, wireless USB, high speed WPAN, wireless sensors networks, wireless telemetry, and telemedicine.

The UWB antenna is a crucial component of such a system. The best choice for fabrication of ultrawideband antennas is on planar technology as it allows easly integration with microwave integrated circuits (MIC) and is light weight and relatively of low cost [3]. However, it is a well-known fact that conventional planar monopole antennas have relatively narrow impedance bandwidth and nonomnidirectional radiation characteristics across a significant portion of the frequency range and require a relatively large surface area. It has been shown that fractal geometries, which are based on space filling and self-similarity attributes, can be used for overcoming some of these deficiencies [4]. Also fractal based antennas can effectively couple energy to free space over their small volume [5, 6]. Furthermore different feeding methodology can be applied on fractal antennas without degrading its performance, for example, microstrip lines [6] and coplanar waveguide (CPW) [7]. CPW transmission line feeding method is popular because of lower loss, low radiation leakage, more conveniece with shunt and series connection on the same side of substrate, and reliance on height of substrate for characteristic impedance [7].

In this letter, a novel and very small CPW-fed UWB antenna design is proposed consisting of a fractal patch resembling specific structure. To extend the antenna's impedance bandwidth the ground-plane is truncated at the middle with a pair of squared-shaped notches and rectangular notches are cut out from its sides. These notches affect the antenna's upper and lower band edge frequencies, thus controlling the antennas impedance bandwidth. It is also shown that by increasing the number of fractals new resonances can be generated that enhance the antenna's impedance bandwidth.

## 2. Monopole Antenna Design

**Figure 1:**
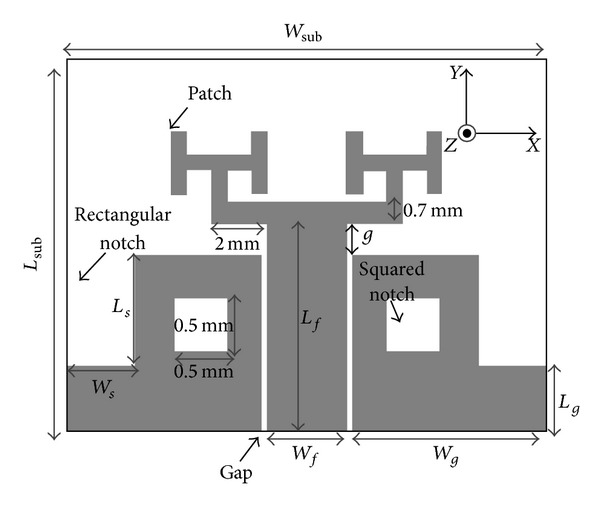
Configuration of the proposed patch antenna with CPW-fed structure (Ant. II).

The configuration and parameters of CPW-fed fractal antenna is shown in Figure 1. The antenna are etched on commercial dielectric substrate FR4 with a thickness of 1 mm, tan*δ* of 0.024, and relative permittivity of 4.4. The substrate dimensions are *W*_sub_ × *L*_sub_. The CPW feedline has width *W*_*f*_ = 3 mm and Gap = 0.3 mm corresponding to a characteristic impedance of 50 Ω. The feedline is tapered for optimum impedance matching the antenna's fractal tree patch.

**Figure 2:**
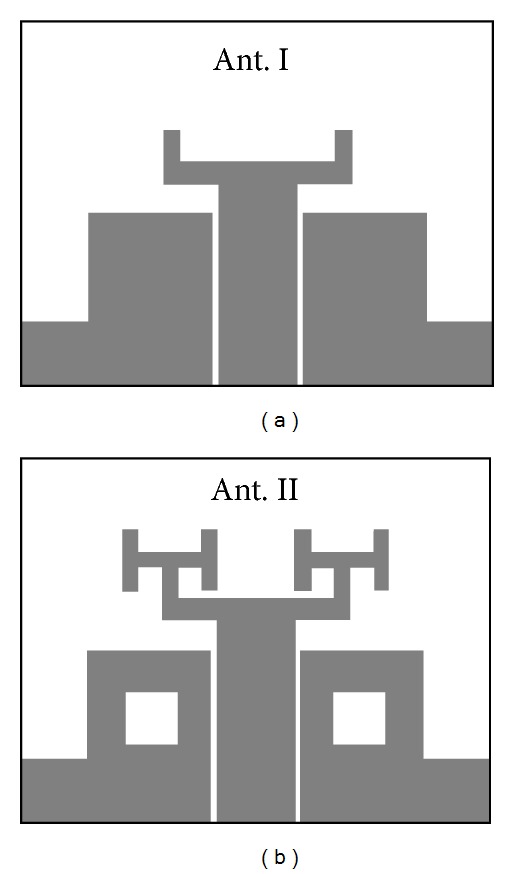
Different antenna configurations (Ant. I and Ant. II).

The fabricated CPW-fed antenna consists of a fractal patch with an array of fractal cells in the form similar to the branches of a tree. The antenna's rectangular ground-plane is printed on the same side as the patch. To achieve a wider impedance bandwidth and hence realize UWB performance notches are cut out from its ground-plane at the sides and centers as shown in Figure 1. The process of antenna modification, illustrated in Figure 2, consists of two steps: firstly, rectangular-shaped notches are cut out from the sides of the ground-plane and secondly each part of ground-plane is truncated with squared-shaped notch at the centers and the fractal patch extended to its second iteration. This modification to the ground-plane enhances the matching characteristics between the patch and the feedline which results in the antenna exhibiting UWB performance. The antenna dimensions were optimized through parametric study using Ansoft's High Frequency Structure Simulator (HFSS). Optimal parameters of the proposed antenna are as follows: *W*_sub_ = 18 mm, *L*_sub_ = 14 mm, *W*_*f*_ = 3 mm, *L*_*f*_ = 7.8 mm, Gap = 0.3 mm, *W*_*g*_ = 7.2 mm, *L*_*g*_ = 4.3 mm, *L*_*s*_ = 2.3 mm, and *W*_*s*_ = 1 mm.

## 3. Simulation and Measurement Results

**Figure 3:**
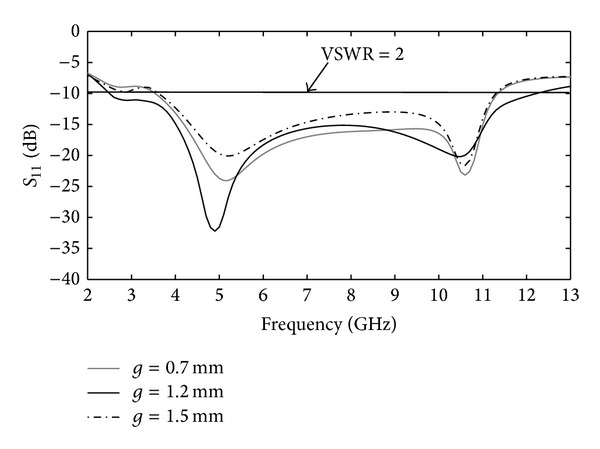
Simulated return-loss response as a function of antenna parameter *g*.

**Figure 4:**
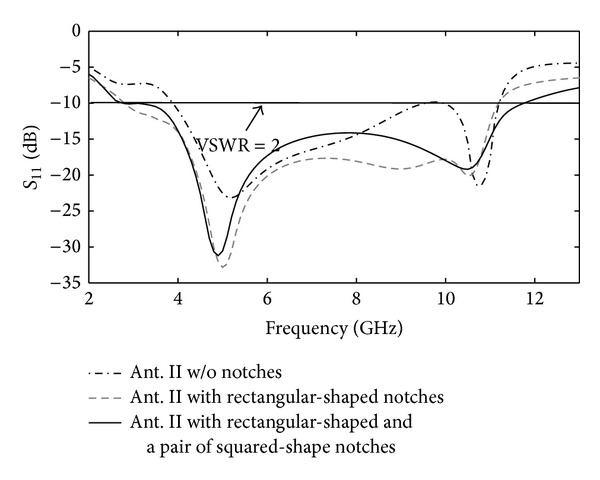
Simulated return-loss response of Ant. II with different ground configurations.

**Figure 5:**
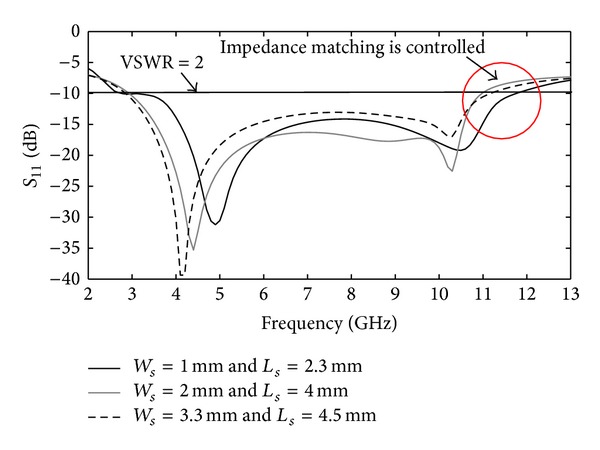
Return-loss response of Ant. II as a function of parameters *W*_*s*_ and *L*_*s*_.

In this section, the parameters of the CPW-fed fractal antenna are discussed, and numerical and experimental results are presented. The antenna's dimensions were determined through the optimization process. The effect of individual parameters was ascertained by changing the parameter in question and keeping all other parameters fixed. The distance between the ground and the fractal patch (*g*) has a major effect on the antenna's impedance matching attributes.  Figure 3 depicts the variation of return loss (*S*_11_) with parameter *g*. The upper and lower values of *g* relative to its optimized value (*g* = 1.2 mm) decrease the operational bandwidth of proposed antenna.  Figure 4 demonstrates the effect on *S*_11_ for different antenna configuration in Figure 2. This figure clearly shows that a pair of squared-shaped notches on ground-plane with rectangular notches at its sides extends the antenna's impedance bandwidth.  Figure 5 shows how the notch dimensions (*W*_*s*_, *L*_*s*_) control the impedance matching of the antenna. It is obvious from Figure 5 that by increasing both parameters from its optimized values (*W*_*s*_ = 1 mm and *L*_*s*_ = 2.3 mm) the matching characteristics deteriorate and the bandwidth reduces correspondingly. The optimum values for *W*_*s*_ and *L*_*s*_ were obtained through parametric study.

The proposed fractal patch is composed of repeating patterns. Increasing the iteration of the fractal patch leads to the generation of strong new resonances that improves the antenna's impedance bandwidth. Antennas realized using one and two fractal iterations were fabricated on printed circuit board. The impedance bandwidth of the two antennas was measured using Agilent 8722ES S-parameter vector network analyzer (50 MHz–40 GHz).

**Figure 6:**
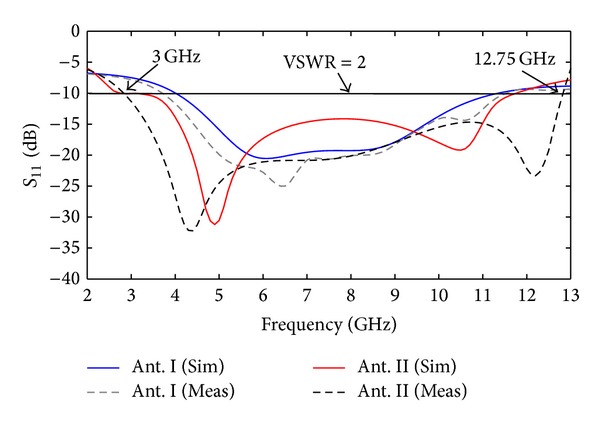
Return-loss response of Ant. I and II.

**Figure 7:**
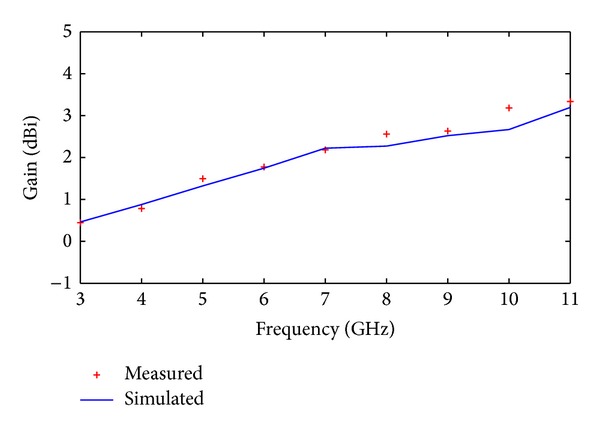
The proposed antenna's measured and simulated gain performance.

Figure 6 shows the simulated and measured response for the two fractal antennas. It is clear that the number of fractal iterations or the number of units has great influence on return loss (*S*_11_). The results show that the second order iteration antenna completely covers the UWB range for *S*_11_ ≤ −10 dB. Measured impedance bandwidth is 9.86 GHz (from 2.95–12.81 GHz) with fractional bandwidth of 125%.  Figure 7 shows the measured antenna gain for Ant. II from 3 to 11 GHz. The measured gain of antenna appears to increase with increment in frequency as predicted by the simulated results.

**Figure 8:**
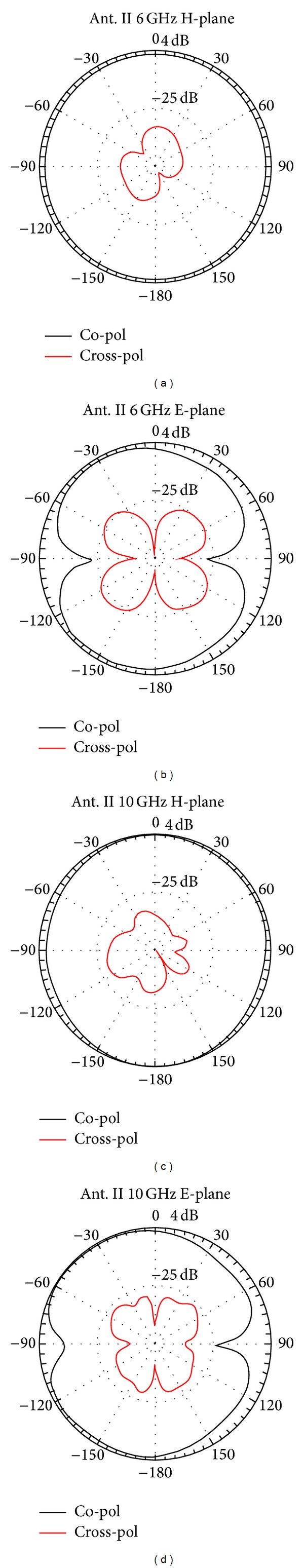
Measured radiation patterns of Ant. II at different frequencies.

The measured radiation patterns of the proposed antenna at 6 and 10 GHz are shown in Figure 8 in the H- and E-planes. The H-plane radiation pattern results show that the proposed antenna is characterized by omnidirectional patterns for all in-band frequencies with cross-polarization down by more than 25 dB. In the E-plane the radiation is bidirectional which reduces with increase in frequency. In this case the cross-polarization is 25 dB down.

## 4. Conclusion 

A compact printed monopole antenna is presented consisting of a fractal radiating patch which is excited with a coplanar waveguide (CPW). It is shown that with the inclusion of two pairs of notches in the ground-plane, one can extend the antenna's performance for ultrawideband applications. The antenna's patch is composed of a definite number of fractal cells. The antenna's parameters were investigated to fully determine the effect on its overall characteristics. Hence, the salient parameters from this analysis enabled the optimization of the antenna's performance. Experimental and simulation results demonstrate that the fractal antenna exhibits desired *S*_11_ level and radiation pattern characteristics across the whole UWB frequency range. The measured results indicate that the antenna operates over a frequency band from 2.95 to 12.81 GHz with fractional bandwidth of 125% for VSWR ≤ 2. The miniaturized antenna has dimensions of just 14 × 18 × 1 mm^3^.
